# Cell‐Membrane‐Inspired Ultrathin Silica Nanochannels Coating for Long‐Term Stable Photoelectrocatalysis with Enhanced Performance

**DOI:** 10.1002/advs.202407686

**Published:** 2024-10-01

**Authors:** Wenyan Yan, Lin Zhou, Zisheng Luo, Shenghua Ding, Dong Li, Xingyu Lin

**Affiliations:** ^1^ College of Biosystems Engineering and Food Science State Key Laboratory of Fluid Power and Mechatronic Systems Zhejiang University Hangzhou 310058 China; ^2^ Institute of Analytical Chemistry Department of Chemistry Zhejiang University Hangzhou 310058 China; ^3^ Hunan Academy of Agricultural sciences Changsha 410125 China

**Keywords:** mechanical stability, photocorrosion, photoelectrocatalyst, pollutant degradation, water splitting

## Abstract

Photoelectrocatalysis has attracted significant attention for water splitting and contaminant degradation. However, the lifetime of photoelectrocatalysis devices is hampered by the severe instability and photocorrosion of the photo‐active nanomaterial on the photoelectrode, which is a key limitation to realizing industrialization. Typically, the conventional protection strategy of photoelectrodes usually suffers from the trade‐off between the photoelectrocatalytic activity and stability. Inspired by biological cell membrane with water channels, here a highly permeable and ultrathin silica coating with ultrasmall straight nanochannels is in situ grown that stabilizes the photoelectrode. These ultrasmall channels boost photoelectrocatalysis by accelerating water transport and reducing the reaction energy within the confined nanochannels. Specifically, the ultrathin coating imparts significant mechanical and structural stability to the photo‐active nanomaterial, thereby preventing its detachment, dissolution, and crystal damage without compromising performance. As a result, the protected photoelectrode exhibits enhanced water splitting activity and excellent stability over 120 h, whereas the photocurrent of the unprotected photoelectrode degrades rapidly. Meanwhile, the coated photoelectrode also exhibits superior photoelectrocatalytic degradation efficiency (>97%), even after the 10th cycle. This strategy is facile and universal and can be extended to construct other stable and high‐performance electrodes for promoting photoelectrocatalysis in practical applications.

## Graphical abstract

Inspired by biological cell membrane with water channels, a highly permeable and ultrathin silica coating nanochannels (uSNC) is in situ grown that stabilizes the photoelectrode. The enhanced PEC activity and stability of uSNC‐protected photoelectrode can be applied for water splitting and photoelectrocatalysis degradation.

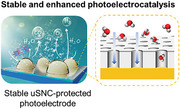

## 1 Introduction

In recent years, energy‐shortage and environmental issues have become popular global topics. Owing to its advantages, which include high energy efficiency, high mass‐energy density, and zero‐carbon emissions, photoelectrochemical (PEC) technology has been widely investigated in the fields of biomass conversion, water splitting, and water purification with the aim of addressing energy and environmental crises.^[^ ^1^ ^]^ Photo‐active nanomaterials play crucial roles in PEC devices, which greatly affect their photocatalysis performance.^[^ ^2^ ^]^ However, these photo‐active nanomaterials on the support are prone to severe detachment, photocorrosion, and destruction during long‐term PEC operation, leading to poor photoelectrode stability.^[^ ^3^ ^]^ In addition, PEC devices applied in industrial reactions must withstand harsh conditions, including continuous stirring and long‐term operation, which pose significant challenges to maintaining photoelectrode mechanical stability.^[^ ^4^ ^]^

Photoelectrodes are modified with photo‐active nanomaterials primarily via weak physical or chemical interactions, including electrostatic adsorption, mechanical interlocking, and van der Waals forces.^[^ ^5^ ^]^ Such weak interfaces lead to poor photoelectrode stability.^[^ ^6^ ^]^ Cover structures have been developed to anchor nanomaterials to their supports and avoid these side effects.^[^ ^7^ ^]^ Common binders include Nafion, graphene oxide, and metal oxide overlayers.^[^ ^8^ ^]^ However, these protective coatings typically limit reactant and product mass transfer, leading to seriously deteriorated PEC performance.^[^ ^9^ ^]^ Usually, the protection strategy of photoelectrode exists a trade‐off between the PEC activity and stability. In a recent study, a hydrogel protective layer was employed on a Pt/TiO_2_/Sb_2_Se_3_ photoelectrode, which improved its mechanical and chemical stability.^[^ ^3c^ ^]^ The hydrogel protector prevented nanoparticle detachment and suppressed dissolution of the photo‐active layer. Although this hydrogel protection strategy resolved stability problems to some extent, hydrogel deformation and rupture have been reported during bubble detachment. Therefore, innovative strategies that stabilize nanomaterial‐based photoelectrodes are required for stable and efficient PEC catalysis.

Cells in organisms are covered by ultrathin cell membranes (5–10 nm), which not only maintain the cell‐structure stability, but also facilitate ultrafast exchange of external molecules in and out of the cell.^[^ ^10^ ^]^ Specifically, an ultrathin membrane ensures that the cellular environment is relatively stable, while the ultrasmall biological channels in the cell membrane (range from 0.5 to 3 nm) exhibit ultrahigh molecular transport with unprecedented fluxes.^[^ ^10^, ^11^ ^]^ It has been reported that short channels and ultrasmall pore are critical for ultrafast mass transport and rapid biochemical reactions.^[^ ^11^, ^12^ ^]^ Thus, similar to the cell membrane, we hypothesized that a biomimetic ultrathin coating with ultrasmall straight nanochannels will enhance both PEC activity and stability.

Inspired by biological cell membrane with water channels, we prepared an ultrathin silica coating (≈9 nm) with vertical‐aligned ultrasmall nanochannels (≈2 nm) to stabilize semiconductor nanoparticles. These ultrasmall nanochannels in the coating accelerate the mass transfer of water molecules and weaken inter‐molecular hydrogen bonds, thereby effectively enhancing photoelectrocatalysis. After coating, the detachment, dissolution, and destruction of photo‐active nanomaterials were reduced even in harsh condition, thereby improving the mechanical and structural stability of the photoelectrode. As a result, the coated photoelectrode exhibits enhanced and stable water splitting, as well as outstanding PEC activity and stability toward degradation.

## 2 Results and Discussion

### 2.1 Preparation and Characterization of the Photoelectrode

**Figure 1:**
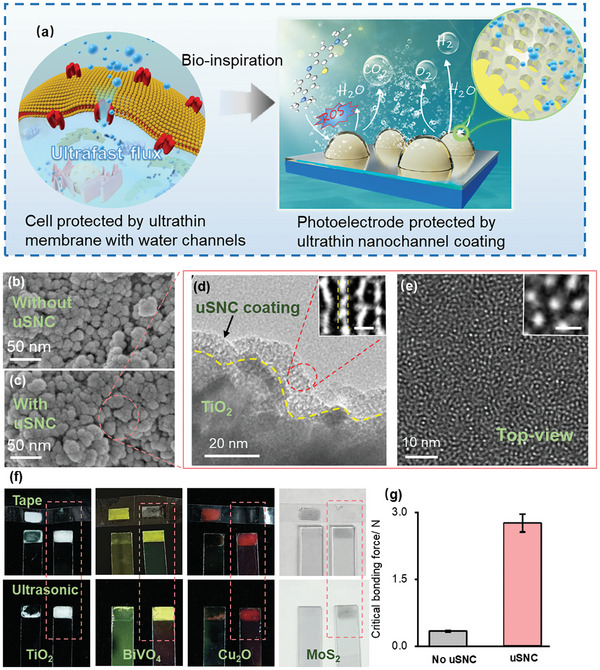
Characterization of the photoelectrode with and without ultrathin nanochannel coating. a) Schematic of uSNC coated photoelectrode for long‐term stable PEC water splitting and dye degradation. b,c) SEM images of photoelectrodes without (b) and with (c) uSNC coating. d) HRTEM cross‐sectional image of uSNC photoelectrode (inset: magnified image with a 5 nm scale bar). e) High‐magnification top‐view TEM image of the uSNC coating (inset: magnified image with a 5 nm scale bar). f) Photographs of diverse photoelectrodes without (left) and with uSNC coating (right) after tape and ultrasonic treatment. The photoelectrode with uSNC coating marked in red box. g) Statistical data of critical adhesive forces of both photoelectrodes. The error bars represent statistical distributions across three samples.

We prepared an ultrathin silica nanochannel (uSNC) coating on top of diverse photoelectrode via in situ bottom‐up growth approach for efficient and stable photoelectrocatalysis (**Figure** **1**a). The detailed growth mechanism of uSNC coating on the photoelectrode surface is shown in Figure S1 (Supporting Information). The growth time was controlled in 5 h to obtain a uniform and ultrathin coating <10 nm. Figure 1b,c shows top‐view scanning electron microscope (SEM) images of synthesized TiO_2_ photoelectrodes with and without uSNC coating (denoted as uSNC and No uSNC, respectively). The nanostructure and morphology of the photoelectrode were essentially unchanged following modification, owing to the ultrathin coating. Meanwhile, the absence of Ti peak in the X‐ray photoelectron spectroscopy (XPS) spectra indicated the uniform and dense coverage of silica‐insert layer onto the whole TiO_2_ photoelectrode surface without any cracks (Figure S2a,b, Supporting Information). The cross‐sectional transmission electron microscopy (TEM) image and corresponding elemental mapping confirmed the presence of an ≈9‐nm‐thick ultrathin coating on the outer surface of the TiO_2_ photoelectrode (Figure 1d; Figure S2c,d, Supporting Information). This uSNC cover contains a high density of ultrasmall nanochannels array perpendicular to the TiO_2_ with a pore size of ≈2.6 nm, as observed in the high‐resolution TEM and the BET method (Figure 1d; Figure S2e, Supporting Information). The vertical‐aligned nanochannel with ultrasmall pore size allows fast water transport in and out of the photoelectrode, similar to biological water nanochannels at cell surface. The nanochannel density was calculated to be as high as 6 × 10^4^ pores·µm^−2^ from the top‐view TEM image, which is an unprecedented value for facilitating transport flux (Figure 1e). The thickness of the coating grown on the photoelectrode can be easily controlled from 5 to 50 nm by simple adjusting the growth time from 4 to 12 h (Figure S3, Supporting Information). The above features clearly indicated that the coating is ultrathin and ultra‐porous with ultrasmall straight nanochannels, which allows for remarkable surface accessibility for efficient photo‐electrocatalysis.

Interestingly, the electrodes covered by uSNC coating exhibited outstanding mechanically stable performance toward protecting the underlying catalyst under harsh conditions. The mechanical stability of photoelectrodes with and without uSNC coating were shown in Figure 1f. The photoelectrode composed of TiO_2_ nanoparticles on ITO glass was easily peeled off, detached, or destroyed by tape and ultrasonic treatment. In contrast, the TiO_2_ nanoparticles were quite stable and did not become detached from the photoelectrode either by tape or ultrasonication after being coated. In addition, the uSNC protector was also successfully applied to other photoelectrodes using diverse nanomaterials, including BiVO_4_, Cu_2_O, and MoS_2_ (Figure 1f; Figure S4, Supporting Information). SEM and HRTEM revealed that the uSNC protector successfully grew in situ on the surface of these photoelectrodes with well‐defined robust interfaces between the uSNC protector and the photo‐active nanoparticles, which helps to retain the mechanical integrities of both materials. The coated BiVO_4_, Cu_2_O, and MoS_2_ photoelectrode were highly stable during treatment with tape or ultrasound, whereas uncoated photoelectrode became completely detached (Figure 1f). Furthermore, a micro‐scratch tester was used to study the critical binding force between the nanomaterial and the electrode. The needle of this tester passes across the surface of the coating with an increasing normal force, causing the coating to begin to detach at a specific normal force (i.e., the critical binding force). The uSNC covered catalyst peels off from the electrode at critical binding force of 2.75 N, while 0.29 N was measured for bare TiO_2_ photoelectrode (Figure 1g; Figure S5, Supporting Information), indicating that the photoelectrode with uSNC protector has stronger interfacial binding force than that the photoelectrode without uSNC coating. All these results demonstrated that the uSNC coating can effectively improve the structure and mechanical stability of nanomaterial‐based photoelectrode. It should be noted that the ultrathin nanochannel protection strategy used in this work is simple, facile, and cost‐effective, and is expected to be suitable for large‐scale photoelectrocatalysis.

### 2.2 PEC Activity of Photoelectrodes

**Figure 2:**
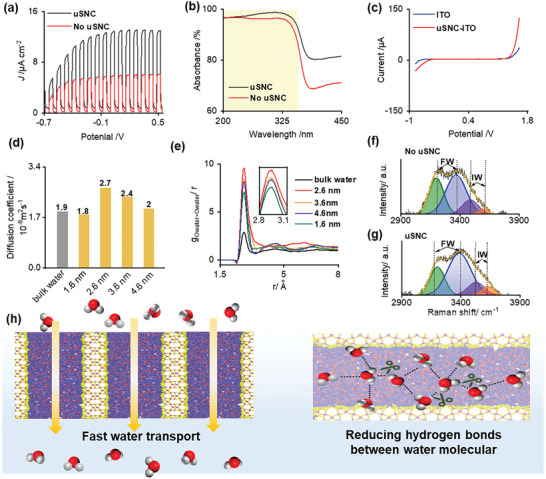
PEC activity of the TiO_2_ photoelectrode with and without ultrathin nanochannel coating. a) Photocurrent density‐potential curve of TiO_2_ photoelectrodes with and without uSNC coating under UV light irradiation. b) UV–vis absorption spectrum of both photoelectrodes. c) Linear sweep voltammogram of ITO electrode with and without uSNC coating in 0.1 M Na_2_SO_4_. d) Simulated diffusion coefficient of water molecules within the uSNC nanochannel with different diameters. e) RDF among oxygen atoms of water molecules within the uSNC nanochannel with different diameters. f,g) Raman spectra and fitting curves in the energy region of wet photoelectrode without (f) and with (g) uSNC coating. h) Schematic of the enhanced PEC activity of TiO_2_ photoelectrode by the nanochannel coating.

The PEC activity of the photoelectrode was evaluated by current density‐potential measurement in a 0.1 M Na_2_SO_4_ solution under simulated UV irradiation. Typically, there exists a trade‐off between the PEC activity and the use of protective coatings, as it may limit mass transfer of reactants and products.^[^ ^3a^ ^]^ Due to the ultrathin material with high density and straight nanochannels, the coating may not affect the photoelectrocatalysis performance. Interestingly, compared to that without protection, the photocurrent density of uSNC photoelectrode was increased from ≈5 µA cm^−2^ to ≈10 µA cm^−2^ at 0 V_RHE_ (**Figure** **2**a), which indicated that the protective uSNC coating provides a viable approach for enhancing the PEC activities of photoelectrodes despite being an insulator. The light‐absorption of photoelectrode was measured by UV–vis DRS spectrum (Figure 2b). Compared to unprotected photoelectrodes, the uSNC photoelectrode showed increased adsorption by ≈5%, which may arise from the anti‐reflective performance of the transparent uSNC film (Figure S6, Supporting Information). Therefore, the optical effect is not the main factor for the enhanced photocurrent. Considering that the top protective uSNC coating is an insulator and the bottom of the electrode contains the same photo‐active materials, we attribute the enhanced photocurrent density to enhanced catalysis reaction inside ultrasmall confined spaces.^[^ ^9a^ ^]^ To provide evidence for this hypothesis, the electrochemical measurement for water oxidation was conducted by coating uSNC on bare ITO glass (uSNC‐ITO) without using photo‐active nanomaterials. In this case, all the photoelectrocatalysis process can be ignored. As shown in Figure 2c, compared with the bare ITO electrode, an enhanced water oxidized was also observed after introducing uSNC coating, indicating that the nanochannel coating indeed accelerate interface water reaction inside nanoconfined spaces.

To gain deeper insight into the reaction at the molecular‐level, the characteristic of water molecules inside ultrasmall nanochannel was studied using the all‐atom molecular dynamics (MD) simulation.^[^ ^12b^ ^]^ As shown in Figure 2d and Figure S7 (Supporting Information), the diffusion coefficients for water molecules in nanochannels with diameters of 1.6, 2.6, 3.6, and 4.6 nm were modeled. An enhanced water diffusion at pore size of ≈2.6 nm was observed, which may be due to fast water transport in the ultrasmall nanochannels caused by capillary‐like pressure and space confinement effects.^[^ ^9^, ^12^, ^13^ ^]^ As the pore size further decreased, the movement of water molecular was hindered significantly by the space restriction, and diffusion coefficient decreased. In addition, the water molecule transport through the nanochannels was further explored using finite element analysis.^[^ ^13^ ^]^ As shown in Figure S7c (Supporting Information), the uSNC layer was simplified into a single nanochannel. The contact angle was set to θ = π/8 to indicate a hydrophilic surface, indicated by water contact angle testing (Figure S8, Supporting Information). The results indicate that these ultrasmall nanochannels also facilitate the fast water transport to the underlying photoelectrode under the action of capillary forces. These results prove that the introduction of the uSNC coating accelerated the mass transport of water molecules, which increases collisions between water molecules and the catalyst, thereby improving the interfacial reaction rate and PEC activity.

Radial distribution functions (RDF) were also calculated to further describe the state of the water molecule within the ultrasmall nanochannels, as shown in Figure 2e. It is observed that water molecules in the nanochannel have enhanced density compared to bulk water, indicating more densely packed water molecules in the ultrasmall nanochannel. Figure S7d (Supporting Information) also showed that the average number of hydrogen bonds between water molecules (O_water_‐O_water_) in the nanoconfined space was reduced. These results indicated that the introduction of ultrasmall nanochannel break ups the strong interaction between water molecules, which reduces the potential energy for water oxidation and enhances PEC performance. The number of hydrogen bonds of water molecules on the photoelectrode with and without uSNC was also experimentally examined by Raman spectroscopy (Figure 2f,g).^[^ ^13^ ^]^ Intermediate‐water: free‐water (IW: FW) ratios with and without uSNC are 0.23 and 0.2, revealing that more water molecules with reduced hydrogen bond were present in the ultrasmall nanochannel. Therefore, it can be concluded that the uSNC coating accelerates water mass transport and reduces the number of intramolecular hydrogen bonds of water in the nanoconfined space, thereby promoting PEC activity of photoelectrodes, as shown schematically in Figure 2h.

### 2.3 PEC Stability of Photoelectrodes

**Figure 3:**
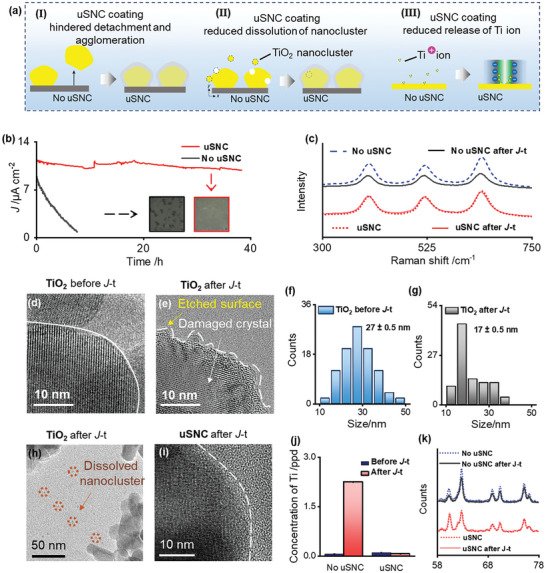
PEC stability of the TiO_2_ photoelectrode with and without ultrathin nanochannel coating. a) Schematic illustration of enhanced stability of the TiO_2_ photoelectrode by uSNC coating. b) Chronoamperometry *J*‐t curves of photoelectrodes with and without uSNC coating (inset: photographic images of both photoelectrodes after long‐term measurement). c) Raman spectra of both photoelectrodes before and after *J*–*t* measurement. d) HRTEM image of photoelectrodes without uSNC coating before *J*–*t* measurement. e,h) HRTEM image of photoelectrodes without uSNC coating after *J*–*t* measurement. f,g) Size distributions of photoelectrodes without uSNC before (f) and after (g) *J*–*t* measurement. i) HRTEM image of photoelectrodes with uSNC coating after *J*–*t* measurement. j) ICP analysis of Ti element in the electrolyte before and after *J*–*t* measurement of both photoelectrodes, the error bars represent the standard deviations of three samples. k) XRD pattern of both photoelectrode before and after *J*–*t* measurement.

The photoelectrode applied in long‐time operation may suffer from severe detachment and photocorrosion of photo‐active nanomaterials, which significantly affects the lifetime and PEC performance of the photoelectrode. **Figure** **3**a shows that the uSNC coating is expected to provide excellent mechanical stability by in situ anchoring the photo‐active nanomaterial onto the substrate, thereby suppressing the detachment of nanoparticles. Meanwhile, the ultrasmall channel with negatively charged surface will hinder the release of Ti ions and Ti nanocrystals caused by photocorrosion. As expected, by employing the protective nanochannel coating, the lifetime of photoelectrode was extended remarkably, as shown in Figure 3b. The *J*–*t* plot of the TiO_2_ photoelectrode coated with uSNC remained stable for 40 h with well‐maintained photocurrent density, whereas the bare TiO_2_ photoelectrode showed a significant initial drop and continued to drop over time, maintaining 10% of the initial photocurrent density value after 9 h. The stability of the as prepared photoelectrode was also confirmed by linear sweep voltammetry (LSV) in Figure S9a (Supporting Information). After long‐time *J*‐t measurement, the photo‐electric performance of the bare electrode decreased rapidly, whereas the LSV curve for the uSNC electrode remained unchanged, indicating excellent long‐term stability of the photoelectrode with uSNC coating.

After long‐time test, the surface of uncoated photoelectrode was destroyed, as shown in the inset of Figure 3b. SEM further observed the detachment of nanoparticles fromphotoelectrode surface (Figure S10a,b, Supporting Information). Meanwhile, the destroyed structure of the photoelectrode was confirmed by Raman spectroscopy and Ti2p XPS spectra with the decreasing intensity of characterize TiO_2_ peak (Figure 3c; Figure S10e, Supporting Information). However, with uSNC protection, the photoelectrode exhibited uniform and unchanging surface without any damage, as shown in Figure 3b,c and Figure S10c–e (Supporting Information), demonstrating that the in situ immobilization of uSNC protective coating will prevent the detachment of nanoparticles. To further verify the robustness of the uSNC photoelectrode, the long‐time stability experiment was also tested after ultrasonic treatment for 3h, the photoelectrode with uSNC still exhibited stable and enhanced photocurrent density during long‐time ultrasonics treatments, as shown in Figure S9b (Supporting Information). These results demonstrated that even in harsh operating conditions, the uSNC coating indeed offer an excellent mechanical stability and superior photoelectrochemical performance.

To further understand the underlying mechanism, we characterized the microscopic morphology of the photoelectrode before and after long‐time measurement via HRTEM analysis. Compared with original TiO_2_ (Figure 3d), the surface of TiO_2_ was etched after test in Figure 3e. The average particle size of TiO_2_ nanoparticles decreased from 27 nm to 17 nm after measurement (Figure 3f,g; Figure S11a,b, Supporting Information). Besides, many small nanoclusters (indicated by red circles) ≈5 nm in size appeared in the electrolyte, indicating that the TiO_2_ nanoparticles undergone severe dissolution caused by photocorrosion during PEC testing (Figure 3h; Figure S10f, Supporting Information). It was mainly because that Ti‐O bonds were destroyed by photo‐holes at the surface lattice with the progress of the PEC reaction, generating small particles and falling off in the electrolyte.^[^ ^14^ ^]^ The dissolution of nanoparticles can be also confirmed by the presence of Ti elements in the electrolyte, as determined by ICP‐MS results in Figure 3j. However, with uSNC, no Ti was observed in the electrolyte after long‐time test, indicating that the detachment and dissolution can be avoided by the in situ grown uSNC coating. Figure 3i shows that the TiO_2_ remained intact without any surface etching. The superior structural stability with uSNC coating is attributed to its ultrasmall nanochannels. On the one hand, the released nanocluster, although small in size, cannot pass through the ultrasmall nanochannel and immobilized in situ by the uSNC coating. On the other hand, dissolved Ti^4+^ ions can be in situ captured by negatively charged ultrasmall nanochannels, thereby suppressing the additional dissolution of the TiO_2_ nanomaterial.^[^ ^3a^ ^]^ Apart from detachment and dissolution, the crystal structure of TiO_2_ nanoparticles was destroyed after measurement (Figure 3e), whereas the crystal structure of uSNC photoelectrode was still identical without any loss (Figure 3i), indicating that the in situ grown of uSNC coating on the photoelectrode surface effectively protects the crystal structure of nanoparticles. XRD measurements and also confirm this result (Figure 3k). The superior stability of the crystal structure is attributable to confinement imposed by the nanoporous protector, which anchors on the surface of TiO_2_ to prevent crystal change. All results indicated that the uSNC effectively prevented the detachment, dissolution, and damage of crystal structure of TiO_2_ during long‐term PEC testing. Recently, several strategies for stabilizing photoelectrodes have recently been developed. However, the diffusivity of reactants and products were severely decreased after modification, which deteriorates the performance factors. Meanwhile, some strategies can only be used only for specific nanomaterials, which is not universal. In contrast, ultrathin nanochannel protective coatings used in this work are facile, simple, and universal, which could enhance both stability and efficiency of PEC.

### 2.4 Versatility of the Ultrasmall Nanochannel Protector

The versatility of the uSNC coating was evaluated by applying it to typical photoanode (BiVO_4_) (Figure S12a, Supporting Information). The Chronoamperometry *J*‐t curve of BiVO_4_ coated with uSNC photoanode showed enhanced photocurrent density and kept an incredible stability for over 5 h. In comparison, an unstable photocurrent density was observed for that without uSNC. Notably, the proposed uSNC protector is applicable to both photoanodes and photocathodes. When the Cu_2_O photocathode was protected by uSNC (Figure S12c, Supporting Information), the photocurrent and lifetime were dramatically enhanced. In addition, the surface structure of these photoelectrode with uSNC coating remained intact even after ultrasonic treatment (Figure S12b,d, Supporting Information), thereby demonstrating the protection of the uSNC coating during PEC operation. The developed ultrasmall nanochannel coating for enhancement and protection strategy is universal and catalyst‐independent, and can generically be adapted in various PEC devices.

### 2.5 Application for Long‐Term Enhanced Water Splitting

**Figure 4:**
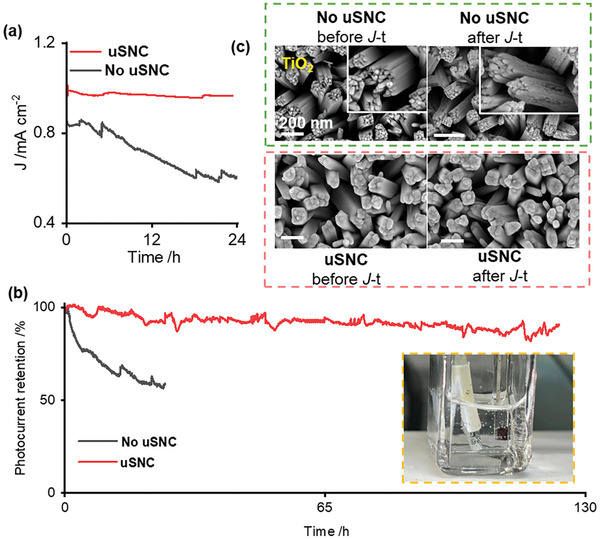
Water splitting of the TiO_2_ photoelectrode with and without the nanochannel coating. a) Chronoamperometry *J–t* curves of the TiO_2_ nanorod‐based photoelectrode with and without uSNC coating at a potential of 1.23 V (vs SHE). b) The photoelectrochemical stability of both photoelectrodes during long‐term test. c) SEM image of both photoelectrodes before and after *J*–*t* measurement.

Constructing stable photoelectrodes for long‐term water splitting is essential. As is well‐known, the generated bubble over the catalyst during water splitting pose significant challenges to long‐term stable operation.^[^ ^15^ ^]^ As a typical photocatalyst, cost‐effective and environmentally benign TiO_2_ nanorods have been adopted as photoelectrodes due to their excellent performance for water splitting. As shown in **Figure** **4**a, the *J*‐*t* plots of the TiO_2_ nanorod without uSNC coating showed an unstable current density ≈0.5 mA cm^−2^ under the continuous illumination. However, after the photoelectrode was coated with uSNC, not only was significant enhanced in current density up to 1 mA cm^−2^ observed, but the stability of the current was also maintained for 120 h (Figure 4b). Furthermore, the photon‐to‐current conversion efficiency (IPCE) and water splitting efficiency was also measured, indicating that more photons can be effectively converted to hydrogen gas with high Quantum efficiency (Figure S13, Supporting Information). In addition, SEM (Figure 4c) and HRTEM diffraction technique (Figure S14, Supporting Information) images after 10 h stability measurement showed that the microstructure and crystal structure of the catalyst without protection was etched and destroyed. In comparison, the catalysts with uSNC coating have no change observed on the surface after long‐term stability measurement, suggesting the excellent protection of uSNC coating.

### 2.6 Application for Long‐Term Enhanced Degradation

**Figure 5:**
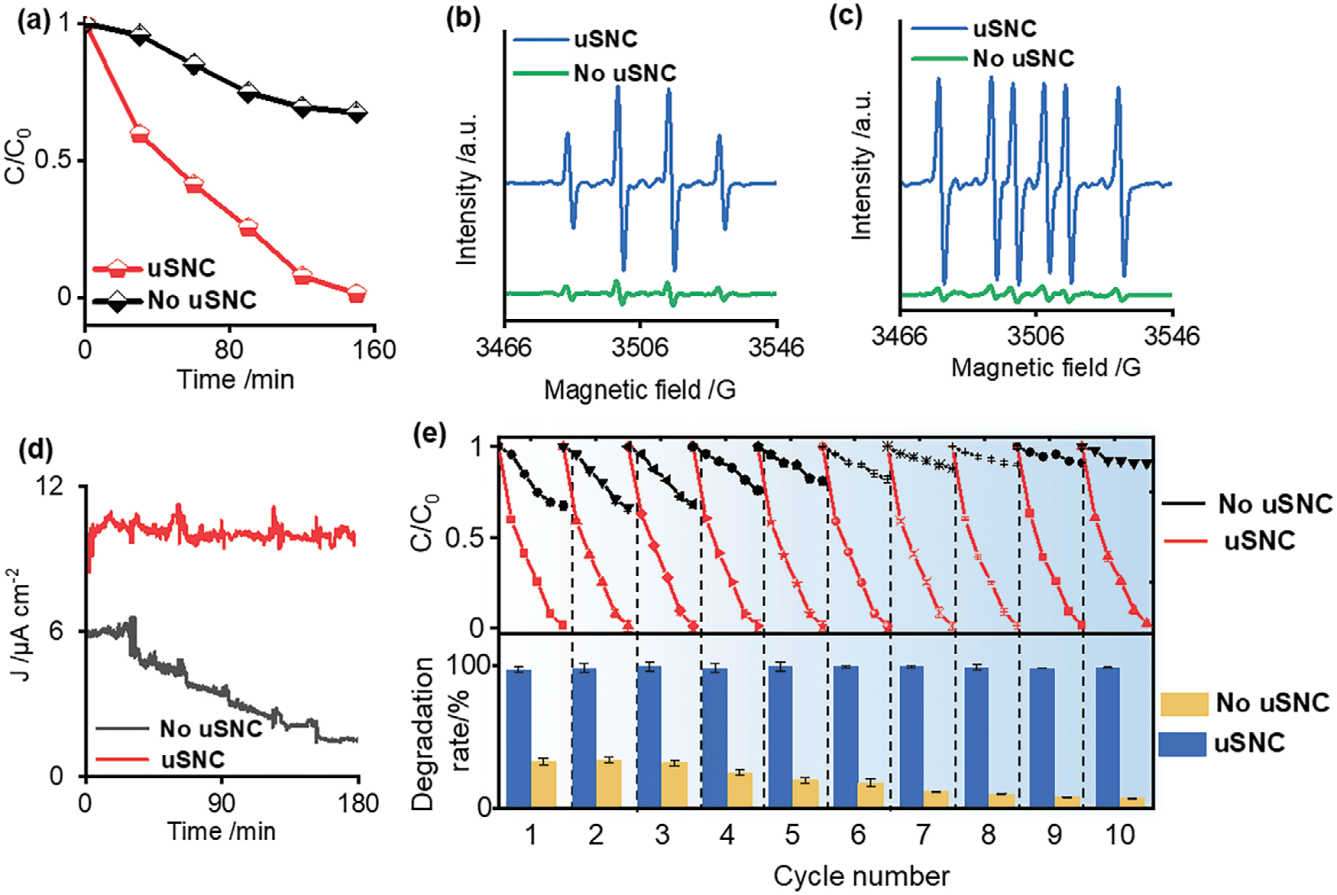
Photocatalytic degradation performance of the TiO_2_ photoelectrode with and without ultrathin nanochannel coating. a) The PEC degradation rate of MB by photoelectrodes with and without uSNC coating (the error bars represent the standard deviations of triplicate measurements). b) ESR spectra of DMPO‐^•^OH adduct. c) ESR spectra of DMPO‐O_2_^•−^adduct. d) Chronoamperometry degradation MB curves of both photoelectrodes. e) The cycle stability of both photoelectrodes (the error bars represent the standard deviations of triplicate measurements).

The photo‐electrocatalytic performance of the obtained photoelectrode after coating was then demonstrated by the long‐term degradation efficiency. As shown in **Figure** **5**a, with the uSNC coating, the degradation efficiency for methylene blue (MB) with an initial concentration of 2 mg L^−1^ was 99.7%, while the pristine TiO_2_ photoelectrode can only decompose 36.3%. Meanwhile, to show the reproducibility of MB degradation, five independently prepared photoelectrodes were evaluated. As shown in the Figure S15a,b (Supporting Information), the photoelectrocatalysis performance and their statistical analysis with and without uSNC coating exhibit significant differences, demonstrating the excellent photoelectrocatalysis performance of the as‐prepared uSNC photoelectrode. Furthermore, the enhanced photoelectrocatalysis performance (along with statistical analysis) also confirmed in the degradation of high concentrations MB (Figure S15c,d, Supporting Information). This enhanced performance is ascribable to the modification of the uSNC coating, which promotes water transport and reduces the number of hydrogen bonds, thereby enhancing water oxidation reaction to generate more reactive oxygen species (ROS). Electron paramagnetic resonance (EPR) spectroscopy further confirmed that large amounts of ^•^OH and O_2_^•−^ were generated with uSNC protection compared with pristine TiO_2_ (Figure 5b,c), indicating that the introduction of uSNC coating enhanced photoelectrocatalysis degradation. More detailed study for MB degradation using the uSNC coated photoelectrode can be found in the Figure S16a–c (Supporting Information).

Figure 5d displays the photocurrent curve during MB photoelectrocatalytic degradation. The photocurrent of the TiO_2_ photoelectrode was observed to decline by 30%, whereas the photoelectrode with uSNC remained stable, only a 3% down in the degradation process, demonstrating the excellent stability of uSNC photoelectrode. Furthermore, as shown in the long‐term cycled degradation experiment (Figure 5e), with the uSNC coating, the photoelectrocatalytic activity of the photoelectrode remained above 97% after ten cycles, while that of TiO_2_ significantly declined from 38% to 7%. Meanwhile, the Ti element was not found in the degradation solution with uSNC coating after ten cycles, which indicates the outstanding stability (Figure S16d, Supporting Information).

## 3 Conclusion

Inspired by ultrathin cell membrane with biological water channels, we developed a facile and cost‐effective protection strategy to significantly enhance both the PEC activity and stability of photoelectrode by introducing ultrathin, ultrasmall silica channel coating. The experimental result revealed that uSNC coating enhanced the PEC reaction kinetics between the photo‐active material and the electrolyte due to fast water transport and low reaction energy within the nanochannel. In addition, the critical adhesive force of the uSNC photoelectrode is nearly 9.48 times higher than that of the unprotected photoelectrode, thereby delivering excellent mechanical stability even in harsh conditions. Consequently, the lifetime of the photoelectrode employing a cost‐effective porous cover as a device‐on‐top protector was substantially high. It could maintain ≈90% of its initial photocurrent, whereas the photo‐active layer without protection exhibited rapid photocurrent degradation. Detailed investigations revealed that the in situ grown uSNC coatings on the surface of the photo‐active nanomaterials effectively inhibit its detachment, while the ultrasmall channel structure of the uSNC coatings reduces the dissolution and changes in crystal structure during the long‐term operation. As expected, the uSNC‐coated photoelectrode exhibited superior PEC activity and durability for over 120h for water splitting. In addition, it exhibited superior photoelectrocatalytic activity and cyclic stability for the degradation of dye under irradiation, with degradation efficiency of over 97% even after ten cycles.

The nanochannel coating enhances insights about the effect of interfacial property on PEC activity and stability of a photoelectrode. Compared to other protection strategy, ultrasmall nanochannel coating developed in this study offers many advantages: i) the uSNC coating effectively enhances both the stability and activity of nanomaterials on photoelectrodes without compromising performance. ii) The uSNC‐protected photoelectrode exhibits superior stability mechanical and structure stability for long time. iii) The protection strategy used in this study is simple and inexpensive. iv) The proposed ultrasmall nanochannel coating for enhancement and protection strategy is universal and catalyst‐independent, and can be generically adapted to various PEC devices. The detailed comparison of our protected photoelectrode with some of the state‐of‐the‐art photoelectrocatalysts is also shown in Table S1 (Supporting Information). Our study addresses these significant issues, maintaining remarkable durability against PEC degradation to facilitate broader usage. Considering the promising stability of our photoelectrodes, future research will explore their potential applications in seawater photoelectrocatalysis.

## 4 Experimental Section

### Fabricating the Photoelectrode

All chemicals were used as received without further purification. The TiO_2_ photoelectrode for photoelectrocatalytic tests was prepared by coating the surface of a 5 cm^−2^ ITO electrode (South China Science &Technology Co., Ltd., Zhuhai, China) with 0.05 mg of TiO_2_ nanoparticles (Xianfeng Nano Material Technology Co., Ltd.; 99.7%; diameter <25 nm, 1 mg mL^−1^) followed by drying at 45°C. The TiO_2_ nanorod for water splitting was produced using a reported hydrothermal method.^[^ ^16^ ^]^ Typically, tetra butyl titanate (0.6 mL) was added dropwise to a 1:1 (w/w) mixture of deionized water and 36–38 wt.% HCl (30 mL), and continuously stirred until transparency was achieved. The mixture was subsequently transferred to a 50 mL Teflon‐lined stainless‐steel autoclave. The FTO electrode was positioned at angle against the Teflon container's wall, with the FTO side facing downward, after which it was heated at 150 °C for 12 h. The TiO_2_ nanorods were cooled, washed with deionized water, and subsequently calcined at 450 °C for 1 h. The 0.05 mg BiVO_4_ and Cu_2_O nanomaterials (Aladdin Chemical Reagent Co., 1 mg mL^−1^) were coated onto the surface of the ITO electrode and dried at 45°C to prepare the BiVO_4_ photoanode and the Cu_2_O photocathode for versatility testing.

### Preparing of the uSNC Coated Photoelectrode

The photoelectrode fabricated with uSNC coating was synthesized using the Stöber solution approach. Firstly, the alkyltrimethylammonium chloride (0.16 g, Aladdin Chemical Reagent Co.,), distilled water (70 mL), ethyl alcohol (30 mL), and ammonia (100 µL, 25–28%) were mixed in a beaker, stirring for 1 h.^[^ ^17^ ^]^ Tetraethyl orthosilicate (80 µL, Aladdin Chemical Reagent Co.,) was then added to the above mixed solution, after which the photoelectrode was immersed in the precursor solution for 5 h using an electrode holder. After dried in an oven at 100 °C for 12 h to obtain uSNC coated photoelectrode.

### Material Characterization

The morphologies of the samples were characterized using SEM (GeminiSEM 300, Carl Zeiss AG, Germany), TEM (JEM‐2010, JEOL, Japan), and HRTEM (2100F, JEOL Electronics, Japan). XPS was performed using a Thermo Scientific K‐Alpha spectrometer (Thermo Fisher Scientific Co., Ltd., USA). UV‐vis diffuse reflectance spectra were obtained using a UV–vis spectrometer (Hitachi, U‐3900H). ICP MS was used to determine the Ti‐ion concentration in the electrolyte. Raman spectra were obtained using a Horiba HR Evolution Raman spectrometer. Particle size analyzer was used to determine the nanomaterials particle size in the electrolyte. The phase and crystal structures of the photoelectrodes were characterized by XRD (Cu Kα radiation, λ = 1.54 Å, Bruker D8 Advance, Germany). A 300 W xenon lamp with different wavelength filters was used to provide monochromatic light for the IPCE determination of the photoelectrode. The concentration of H_2_‐production was analyzed using the GC9790II gas chromatograph (Fuli Analytical Instruments Co., Ltd., Zhejiang, China). Electron paramagnetic resonance (EPR) spectra were recorded on a Bruker ESR Nano spectrometer in water using DMPO as the spin‐trapping agent.

### Photoelectrochemical Tests

Photoelectrochemical testing was conducted using a PEAC 200A instrument (Tianjin Aidahengsheng Science‐Technology Development Co., Ltd., Tianjin, China) and CHI660E electrochemical workstation (Chenhua Instrument Co., Ltd., Shanghai, China). The uSNC photoelectrode, platinum wire, and Ag/AgCl served as the working electrode, counter electrode, and reference electrode, respectively. After achieving a stable dark current condition with a constant current, photocurrents recorded under 365 nm light (intensity: 10 mW cm^−2^). Water splitting was performed in supporting electrolyte of 0.1 M Na_2_SO_4_ solution at constant potentials of 1.23V (vs SHE) using the TiO_2_ nanorod coated with uSNC coating as working electrode with a size of 1 cm^2^, under the 300 W xenon light source ((PLS‐SXE300D, Beijing Perfectlight Technology Co., Ltd) with a 365 nm filter (light intensity: 10 mW cm^−2^); the distance to working electrode was fixed to 7 cm.

Photoelectrocatalytic test was performed using the same device as that used in the water splitting experiments. The as‐prepared 0.5 cm^−2^ photoelectrode was inserted into a 5 mL MB solution (2 mg mL^−1^) in a quartz beaker, containing 0.1 m Na_2_SO_4_ as the supporting electrolyte. Under dark conditions, stir for 30 minutes to achieve adsorption equilibrium. Photoelectrocatalytic testing was carried out under the 300 W xenon light source with a 365 nm filter (light intensity: 10 mW cm^−2^) at a constant potential of 0 V. After initiating photoelectrocatalytic process, 200 µL of the pollutant was collected at given time intervals by pipette to determine the residual dye concentration according to the UV absorbance of MB at 664 nm. In addition, a pseudo‐first‐order reaction model was applied to further analyze the experimental data using the following equation:

(1) $$\ln\left(C_{0}/C_{t}\right)\;=\;k\cdot t$$

where, k, C_0_, t, and C_t_ denoted the rate constant, the initial concentration of the organic contaminants, reaction time, and the concentration of the remaining organic contaminants at time ‘t’, respectively.

### Versatility Testing

Versatility testing was performed using the same device as photoelectrocatalytic test under 300 W xenon light with a 400 nm cut‐off filter. The uSNC coating was in situ grown on the BiVO_4_ surface for 6 h to obtain uSNC photoanode and the photocurrent was measured at 0.7 V. The uSNC coating was in situ grown on the Cu_2_O surface for 12 h to obtain uSNC photocathode and the photocurrent was measured at ‐0.4 V.

### Statistical Analysis

Statistical analyses were performed using OriginPro 2021. All data are presented as means ± standard deviations (SDs) from five samples, excluding data from failed experimental groups. Statistical significance was determined using the two‐tailed unpaired Student's t‐test with statistical significance set to: *p* < 0.05 and results indicated as *p < 0.05, **p < 0.01, ***p < 0.001 and no significance (n.s., *p* > 0.05).

### Finite Element Method

Water molecules mass transfer in nanochannel was modeled using the finite element approach in COMSOL Multiphysics.^[^ ^13^ ^]^ The individual nanochannels was defined with the length (L_n_) of 10 nm, and the diameter (D_n_) of 2.6 nm. The movement of the liquid was controlled using the Cahn–Hilliard equation:

(2) $$\frac{\partial \varphi }{\partial t}+u\cdot \frac{\gamma^{\lambda }}{\varepsilon^{2}}\nabla \Psi$$

(3) $$\Psi =-\nabla \cdot \varepsilon^{2}\nabla \varphi +\left(\varphi^{2}-1\right)\varphi$$

where **u** represents the fluid velocity (m s^−1^), γ denotes the mobility (m^3^·s/kg), *λ* is the mixing energy density (N), ε (m) is the interface thickness parameter and *Ψ* is known as the phase field auxiliary variable. The following equation shows the relationship between the mixing energy density, the interface thickness, and the surface tension coefficient:

(4) $$\sigma \;=\frac{2\sqrt{2}\lambda }{3\varepsilon }\;$$

where, the density (kg m^−3^) and viscosity (Pa·s) of the mixture are defined by the following equation:

(5) $$\rho \;=\rho_{w}\;+\left(\rho_{air}-\rho_{w}\right)V_{f2}$$

(6) $$\mu \;=\mu_{w}\;+\left(\mu_{air}-\mu_{w}\right)V_{f2}$$

The subscripts *w* and air represent the single‐phase water and air, while V_f2_ represents the volume fraction of water. The mass transport and momentum in the fluids were characterized by the Navier‐Stokes equation:

(7) $$\rho \frac{\partial u}{\partial t}-\nabla \cdot \left[-pI+\mu \left(\nabla u+{\left(\nabla u\right)}^{T}\right)\right]+\rho u\cdot \nabla u\;=F_{\mathrm{st}}\;$$

(8) −∇·u=0

where *ρ* represents the density (kg/m^3^), µ indicates the dynamic viscosity (Ns/m^2^), p stands for the pressure (Pa). Surface tension (Fst) is introduced into the model to incorporate capillary forces:

(9) $$F_{st}=\;G\nabla \varphi$$

(10) $$G\;=\;\lambda \left[-\nabla^{2}\varphi +\frac{\varphi \left(\varphi^{2}-1\right)}{\varepsilon^{2}}\right]\;=\frac{\lambda }{\varepsilon^{2}}\;\Psi$$

where φ represents the phase field parameter, and G denotes the chemical potential (J/m^3^).

### Molecular Dynamics Simulation

The molecular‐level state of the water in silica nanochannel was carried out using the large‐scale atomic/molecular massively parallel simulator (LAMMPS) software package.^[^ ^12e^ ^]^ Figure S7 (Supporting Information) shows the model consisting of two silica reservoirs connected via silica nanochannels. The left reservoir is ≈70 Å in length and ≈86 Å in height while the right reservoir is ≈20 Å in length and ≈86 Å in height. The silica nanochannel has a length of roughly 66 Å (40 silica units). The O‐H bond of water molecules is 1.0 Å, and the H‐O‐H angle is 109.47°. The charges on the oxygen and the hydrogen sites were set to −1.0484 e and +0.5242 e, respectively. All atoms in the model were assumed to be static. The Van der Waals interactions between atoms were described by a Lennard‐Jones (LJ) potential with parameters ε_o‐o_ = 0.1553 kcal/mol and δ_o‐o_ = 3.166 Å. The Si‐O interaction was calculated according to the modified embedded‐atom method of the interatomic potential. The cut‐off distance for all LJ potentials was set to 10 Å. The study set the LJ parameters for interactions that involve hydrogen atoms to zero, and simulations were performed at T = 300K.

Periodic boundary conditions were used in the out‐of‐plane direction to account for the dimensionality. A specific number of water molecules was initially placed in the left reservoir, with both the capillary and right reservoirs remaining empty. A water molecule passing through the channel disappears from the reservoir on the right.

## Conflict of Interest

The authors declare no conflict of interest.

## Supporting information



Supporting Information

## Data Availability

The data that support the findings of this study are available from the corresponding author upon reasonable request.
