# Supplementary material for: Cell‐Membrane‐Inspired Ultrathin Silica Nanochannels Coating for Long‐Term Stable Photoelectrocatalysis with Enhanced Performance
Source: Adv Sci (Weinh). 2024 Oct 1;11(44):2407686. doi: 10.1002/advs.202407686 (PMC11600248; doi:10.1002/advs.202407686)
Supplement: Supplementary file 1 — Supporting Information [file ADVS-11-2407686-s001.docx]

**Supporting Information**

**Cell-Membrane-Inspired Ultrathin Silica Nanochannels Coating for Long-****Term Stable Photoelectrocatalysis with Enhanced Performance**

Wenyan Yan, Lin Zhou, Zisheng Luo, Shenghua Ding, Dong Li, Xingyu Lin*

Wenyan Yan, Zisheng Luo, Dong Li, Xingyu Lin*

College of Biosystems Engineering and Food Science, State Key Laboratory of Fluid Power and Mechatronic Systems, Zhejiang University, Hangzhou 310058, China

E-mail: xingyu@zju.edu.cn

Lin Zhou

Institute of Analytical Chemistry, Department of Chemistry, Zhejiang University, Hangzhou 310058, China

Shenghua Ding

Hunan Academy of Agricultural sciences, Changsha 410125, China

SI-1. The IPCE was calculated by the following equation^[1]^

IPCE =$\frac{number of incident electrons}{number of absorbed photons}$=$\frac{I_{ph}}{e}\times\frac{ћc}{\lambda P}$ (1)

Where $I_{ph}$ is the generated photocurrent, $e$ is the elementary charge, $ћ$ is the Planck constant, $c$ is the speed of light in vacuum, $\lambda$ is wavelength number and $P$ is the laser power. Faradaic efficiency was calculated by the following equation.

Faradaic efficiency =$\frac{number of reacted electrons}{number of incident electrons}$

=$\frac{2\times number of evolved H2 molecules}{number of incident electrons}$×100%

=$\frac{e_{products}\times n_{products}\times N}{I_{ph}\times/n}$ (2)

Where $e_{products}$ represents the number of electrons required to reduce H_2_O molecule to H_2_ products (*e*=2), $n_{products}$ indicates the productivity of products, $N$ epresents Avogadro’s constant (N = 6.02 × 10^23^), and *n* indicates the elementary charge (*e* = 1.602 × 10^−19^ C). The quantum efficiency (QE) was calculated by the following equation.

QE = $\frac{number of reacted electrons}{number of absorbed photons}$

= IPCE×Faradaic efficiency×100% (3)


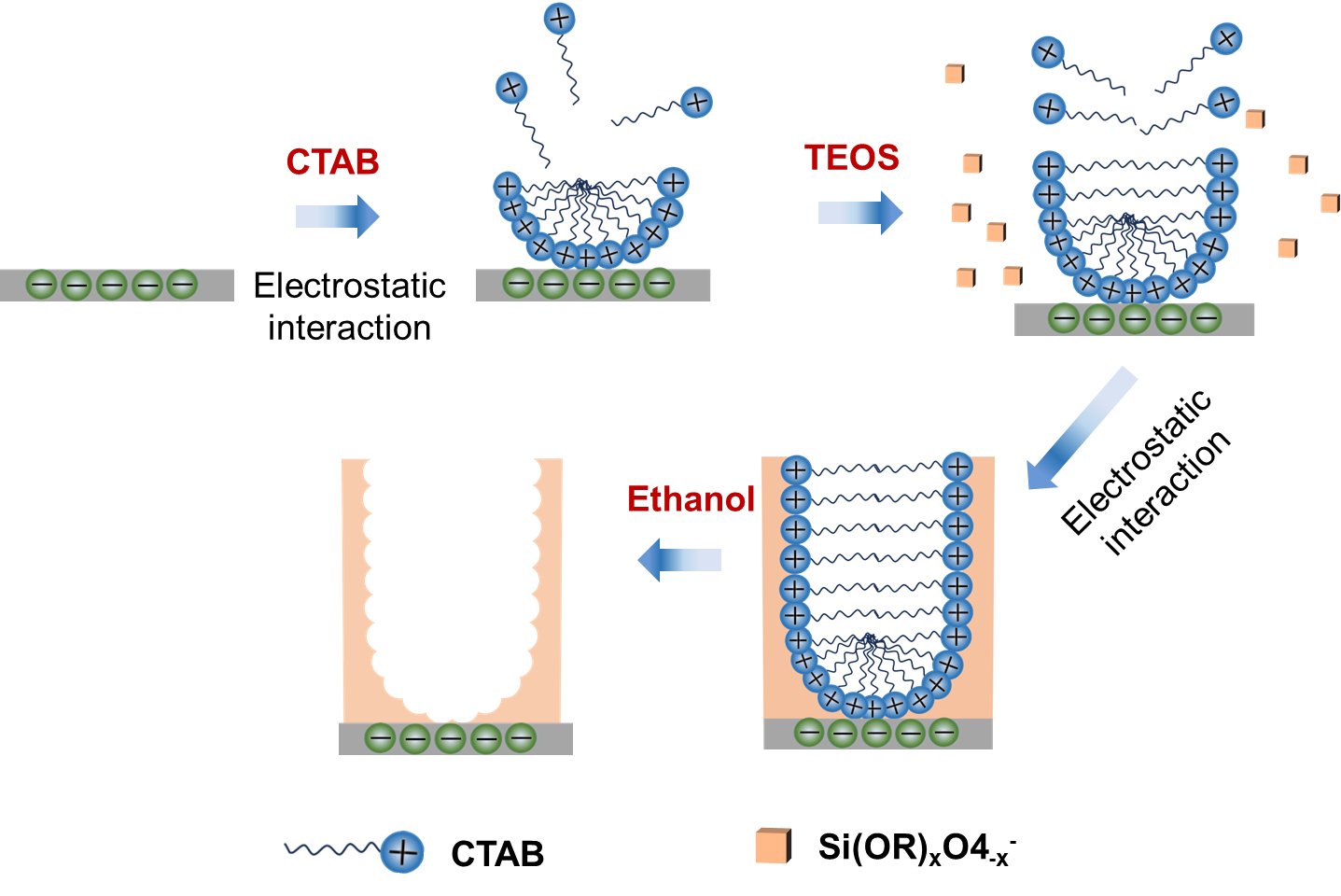


**Figure S1**. Illustration of the formation process of uSNC coating on the TiO_2_ photoelectrode. Briefly, in alkaline electrolytes, metal oxides and related compound are negatively charged due to surface hydroxylation. The surfactant cations (CTA^+^) adsorb onto the negatively charged metal oxides through electrostatic interactions to form spherical micelles. Simultaneously, the silica precursor hydrolyzes to form positively charged oligomeric silicates that adsorb around the CTAB. As a result, the silica film grows vertically on the surface of metal oxides. The ultrathin silica nanochannels coating was obtained by removing the surfactant through solvent extraction.

**
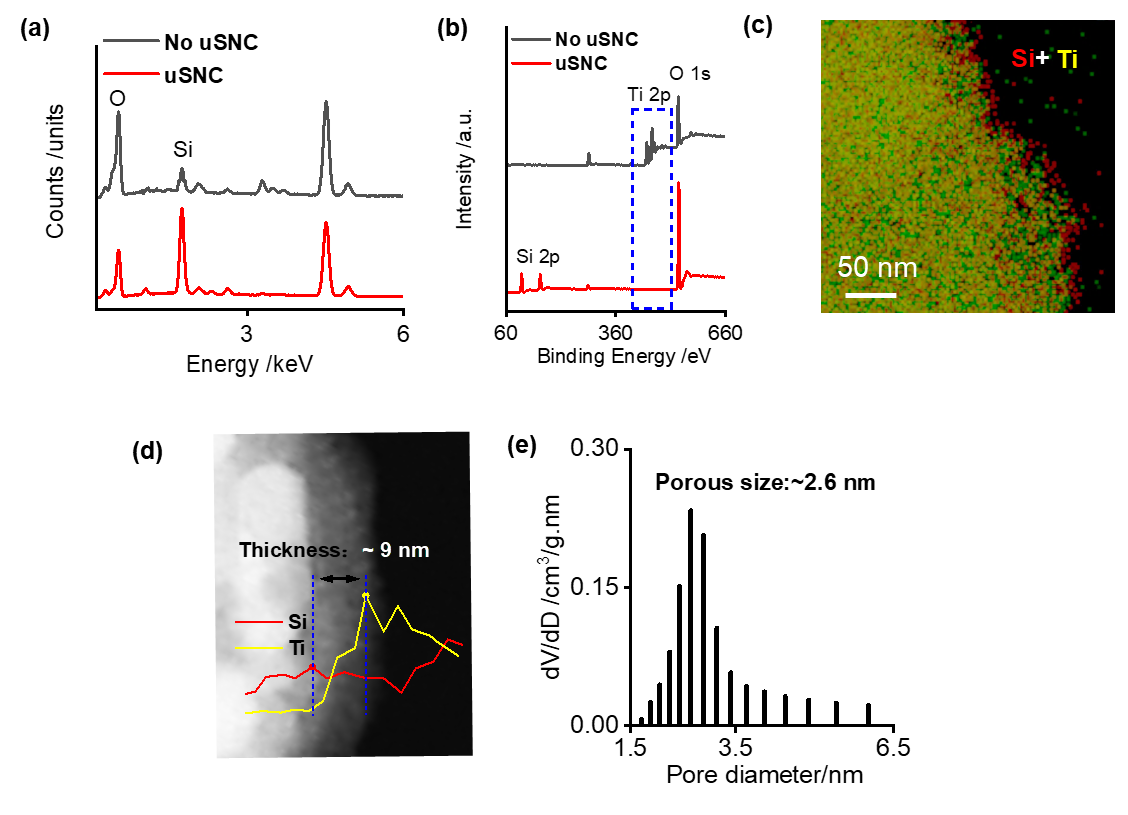
**

**Figure S2.** (a) EDS spectra of TiO_2_ photoelectrodes without and with uSNC coating. (b**)** XPS spectra of TiO_2_ photoelectrodes with and without uSNC coating. The Ti element of the TiO_2_ photoelectrode was disappeared after coated uSNC, indicating that the uniform and dense coverage of silica-insert layer onto the whole TiO_2_ photoelectrode surface without any cracks. (c) EDS mapping of Si and Ti elements in the cross-section of uSNC photoelectrode. (d) Line scan profiles of cross section of uSNC photoelectrode. (e) Pore size distribution curves of uSNC photoelectrode measured by BET nitrogen adsorption/desorption technique.

**
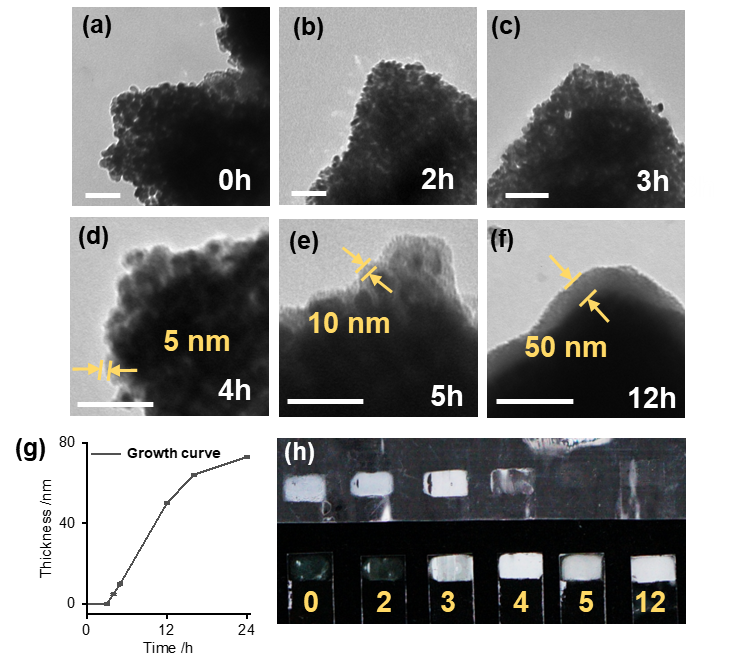
**

**Figure S3.** (a-f) TEM image of uSNC photoelectrode with different thicknesses of uSNC coating (scale:100 nm). (g) Growth thickness of uSNC coating on TiO_2_ surface over time，the error bars represent the standard deviations of three samples. (h) Photographs of photoelectrodes protected by uSNC coating with different thicknesses after tape treatment. The number represent growth time. Result indicates that the uSNC coating of 5 nm will effectively stabilize the photoelectrode.

**
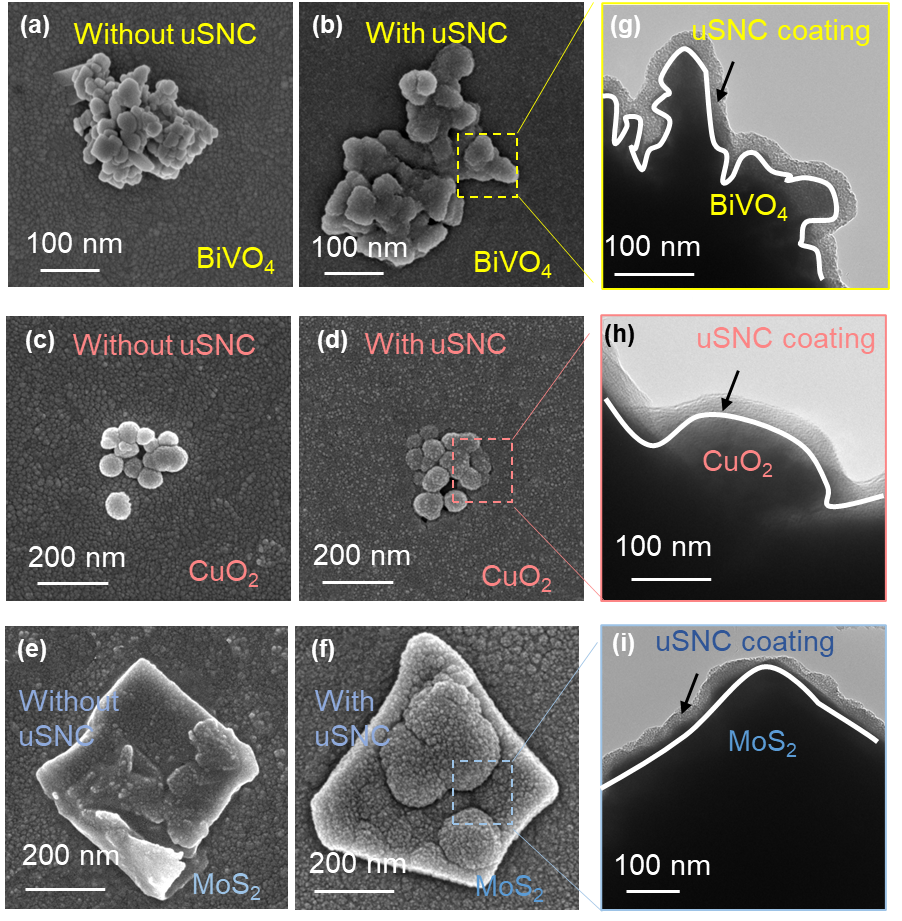
**

**Figure S4.** (a-f) SEM image of BiVO_4_, Cu_2_O and MoS_2_ photoelectrode without and with uSNC coating. (g-i) High-magnification top-view TEM image of the BiVO_4_, Cu_2_O and MoS_2_ with uSNC coating.


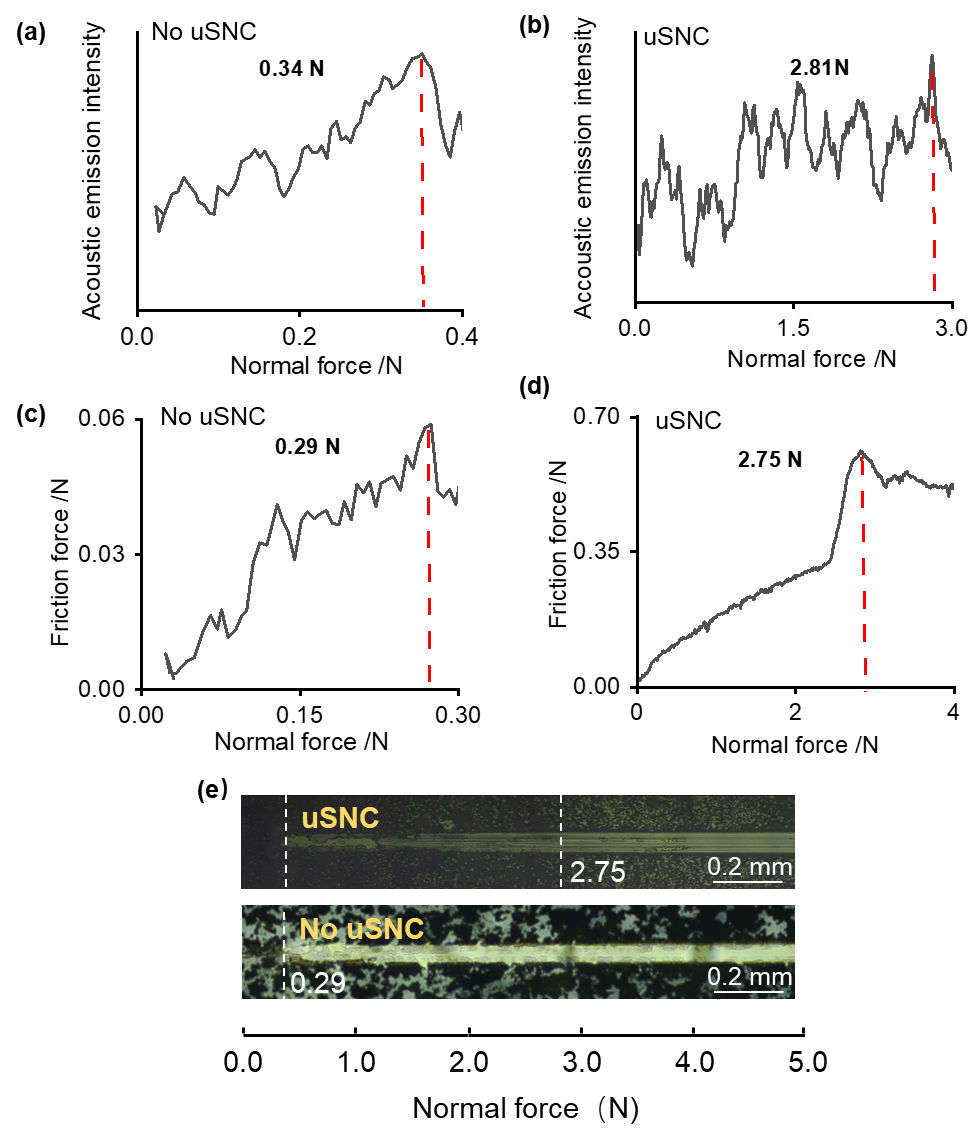


**Figure S5.** (a, b) The friction force-normal force curves of photoelectrodes without (a) and with (b) uSNC coating. (c, d) Acoustic signals of photoelectrodes without (c) and with (d) uSNC coating. (e) The photographs of micro-scratches of photoelectrodes with (top) and without (bottom) uSNC coating. The dotted lines represent the critical adhesive forces corresponding photoelectrode.

We study the critical binding forces of as-prepared catalysts and supports by micro scratch tester.^[2]^ The needle passes across the surface of the coating with an increasing normal force and the coating will begin to peel off at a certain normal force, which is defined as critical binding force. The tested results are confirmed by optical microscopy, friction-normal force curves and acoustic signals (**Figure S4**). The uSNC coated catalyst peels off from the support at a load of 2.75 N while 0.29 N for bare TiO_2_, indicating that the uSNC protected photoelectrode has stronger interfacial binding force than the bare TiO_2_ photoelectrode.

**Figure S6.** Optical reflectance of ITO glass with and without uSNC coating in a wavelength range of 300–700 nm.

We grew the uSNC coating onto the ITO glass and measured the reflectance of those samples without and with uSNC films. ITO glass coated with the uSNC film of 10 nm has the enhanced transmittance, which may be caused by the anti-reflection capability of the coating. The enhanced transmittance of the uSNC anti-reflection coating will effectively enhance the light absorption of photo-active nanomaterials.^[3]^

**
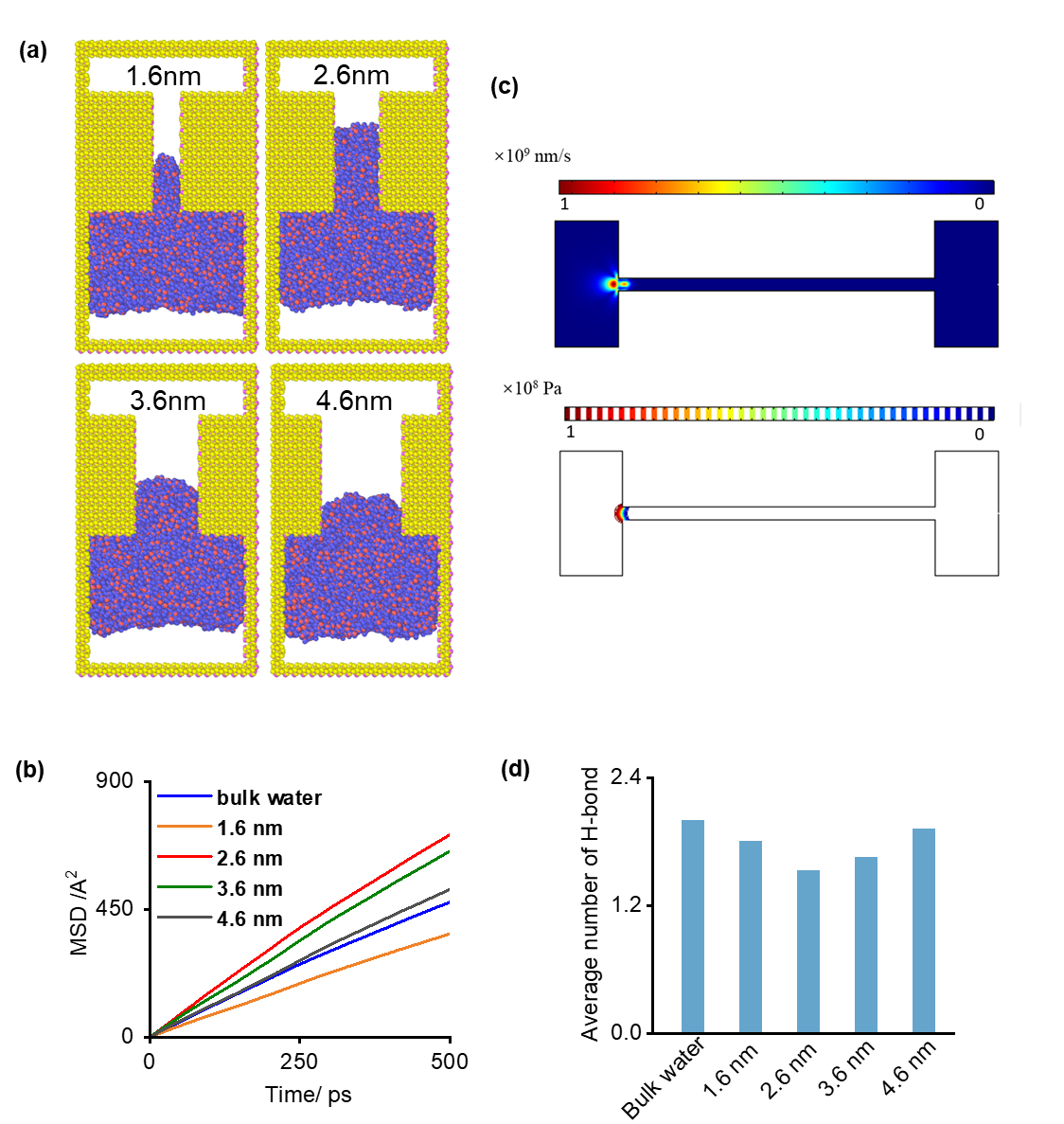
**

**Figure S7.** (a) Model of water-molecule diffusion in uSNC nanocapillaries at 100 ps. (b) MSD of water molecules in the uSNC nanochannel with different diameters. (c) Mass transfer process of the uSNC nanochannel with channel diameter 2.6 nm induced by capillary force from finite element simulations, where the high velocity corresponds to the entrance of water flow and pressure distribution. (d) Average numbers of hydrogen bonds between water-water in nanochannels with different diameter.

**
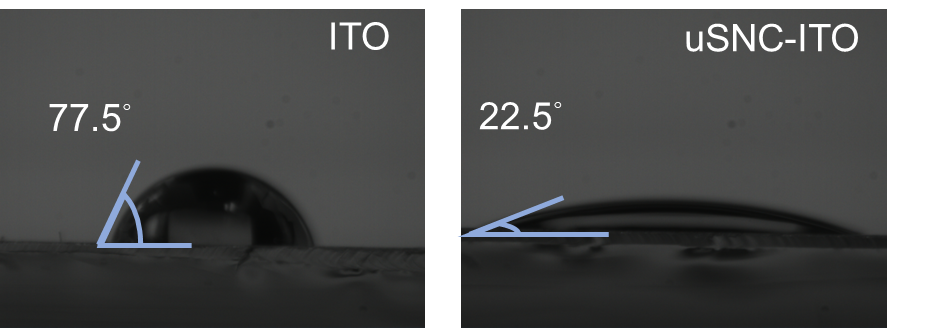
**

**Figure S8.** The water contact angle measurement of ITO electrode with and without uSNC coating. The hydrophilic nanochannel will facilitate mass transfer of water molecules.


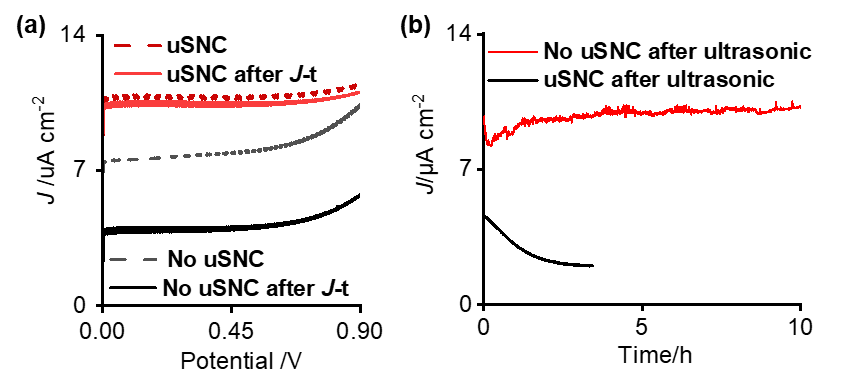


**Figure S9.** (a) *J*-V curve of photoelectrodes with and without uSNC coating before and after *J*–t measurement measured in Na_2_SO_4_ solution. (b) Chronoamperometry J-t curve of photoelectrode without and with uSNC coating after ultrasonic treatment.

It should be noted that compared with untreated photoelectrode, the protected photoelectrode exhibits excellent stability after 3 hours of ultrasonic treatment with unchanged photocurrent density, while the unprotected photoelectrode showed a decrease photocurrent density after only 3 min of ultrasonic treatment and exhibited unstable performance during the *J*–t measurement, indicating that the uSNC-protected photoelectrode has excellent mechanical stability.


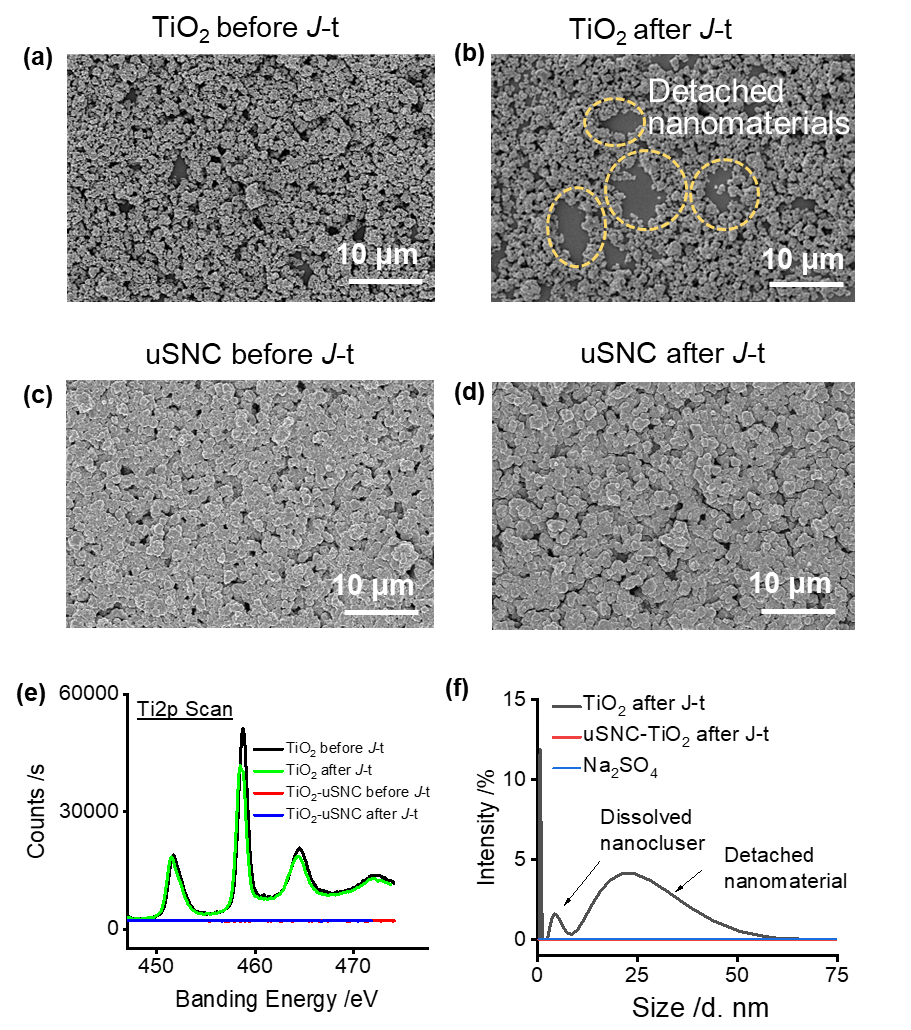


**Figure S10.** (a, b) SEM image of TiO_2_ photoelectrodes before (a) and after (b) *J*-t measurement. (c, d) SEM image of TiO_2_ photoelectrodes with uSNC coating before (c) and after (d) *J*-t measurement. (e) The Ti2p scan of as-prepared photoelectrode before and after *J*-t measurement. The uSNC photoelectrode surface showed no Ti peak after long-term *J*-t testing, indicating that the uSNC coating is still intact without any cracks. (f) The size of dissolved and detached nanomaterials in the electrolyte after *J*-t measurement. The electrolyte of unprotected-photoelectrode contained dissolved nanoclusters of ~5 nm as well as detached nanoparticles of 20-50 nm after long-term testing. However, with uSNC, no nanoclusters were found in the electrolyte after long-time test, indicating that the detachment and dissolution can be avoided by in-situ growth of uSNC coating.


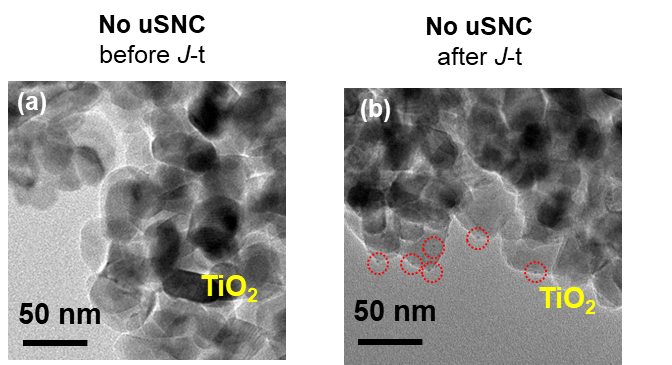


**Figure S11.** (a-b) HRTEM image of TiO_2_ photoelectrode before (a) and after (b) *J*–t measurement.


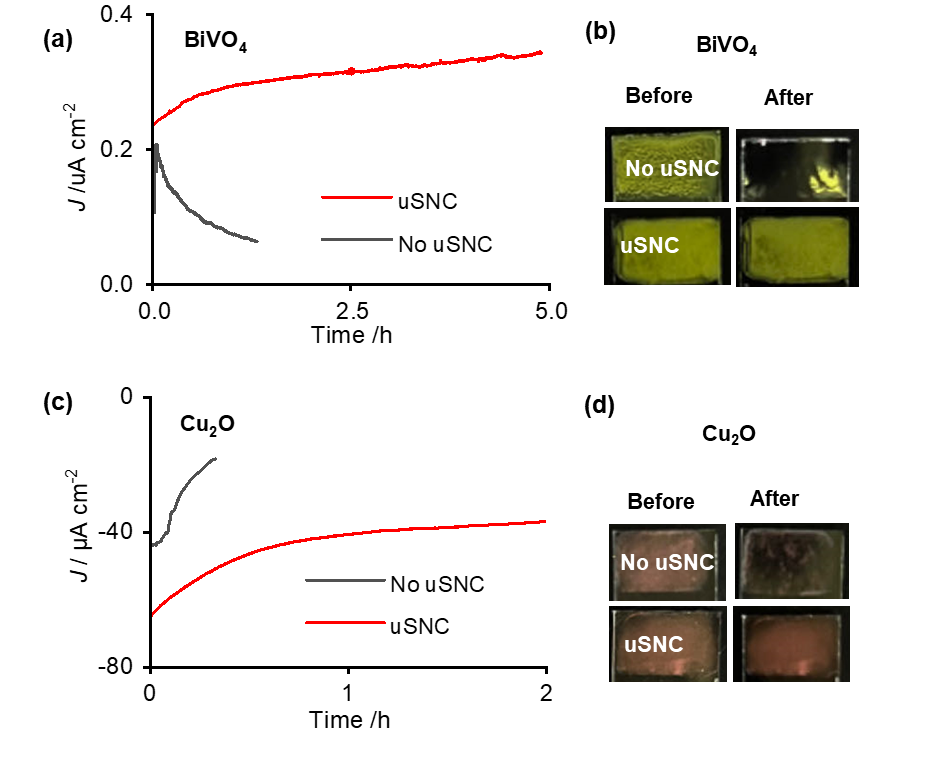


**Figure S12. Versatility of the nanochannel coating.** (a) Chronoamperometry *J*-t curve of the BiVO_4_ photoelectrode with and without uSNC coating in 0.1 M Na_2_SO_4_. (b) The photograph of the BiVO_4_ photoelectrode with and without uSNC coating before and after ultrasonic treatment. (c) Chronoamperometry *J*-t curve of the Cu_2_O photoelectrode with and without uSNC coating in 0.1 M Na_2_SO_4_. (d) The photo of the Cu_2_O photoelectrode with and without uSNC coating before and after ultrasonic treatment.


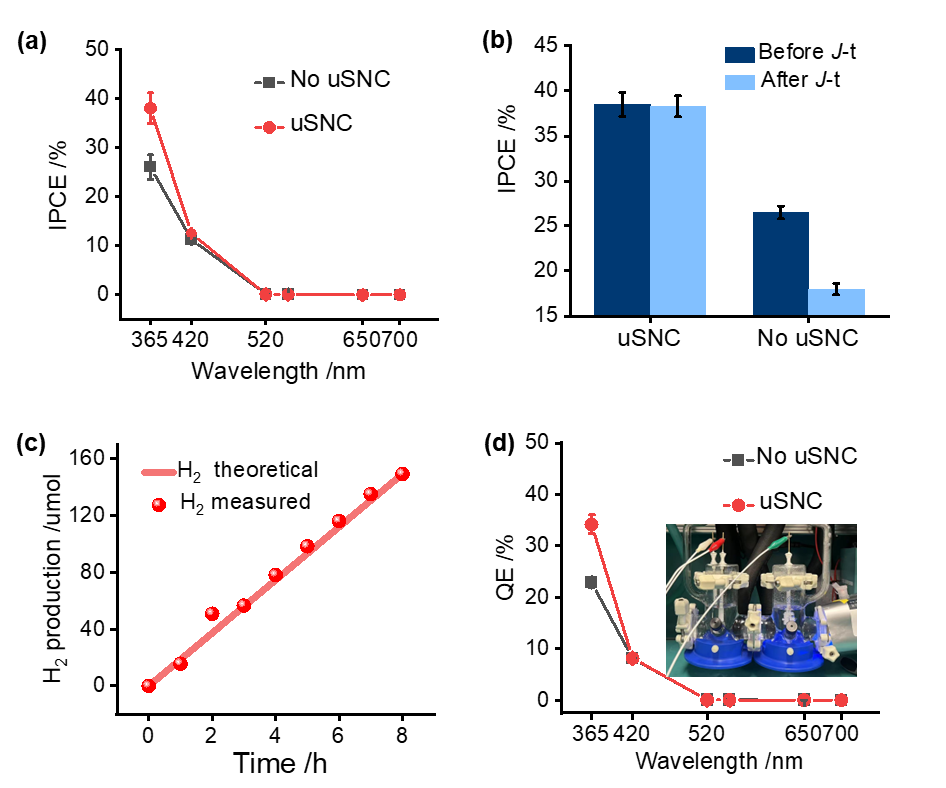


**Figure S13.** (a) IPCE of TiO_2_ photoelectrode with and without uSNC coating at different wavelengths under 1.23 V for 1 h. (b) IPCE of as-prepared photoelectrode before and after *J*-t measurement for 24 h. (c) H_2_-production during stability testing of uSNC photoelectrode. (d) Quantum efficiency for H_2_-production activity of TiO_2_ photoelectrode with and without uSNC coating.


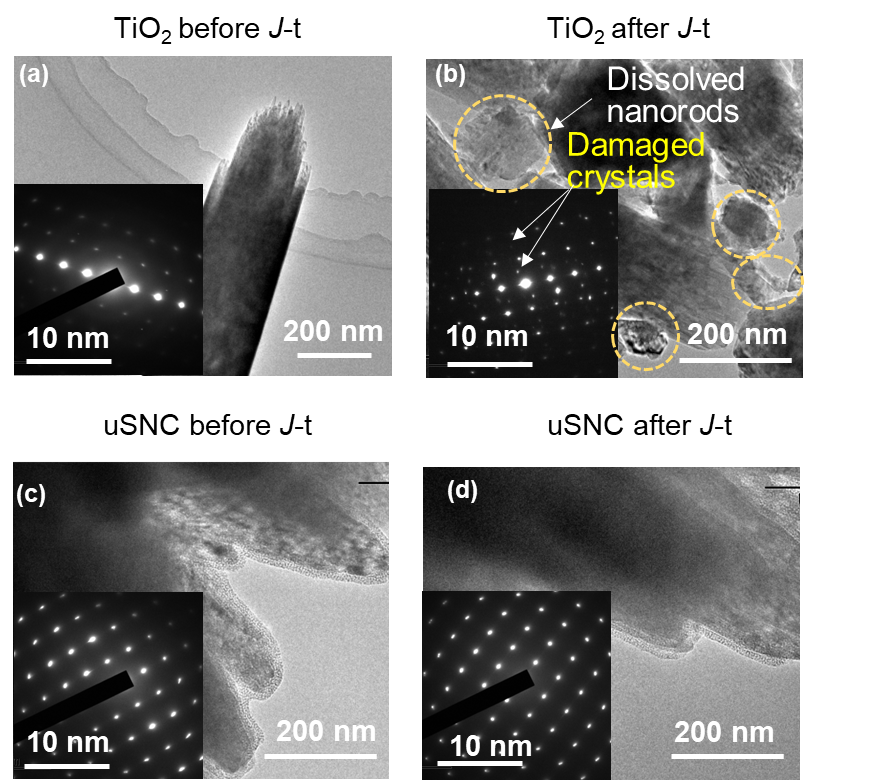


**Figure S14**. (a, b) HRTEM image of TiO_2_ nanorod-based photoelectrodes before (a) and after (b) *J*-t measurement (inset: the corresponding electron diffraction pattern of as-prepared photoelectrode). (c, d) HRTEM image of TiO_2_ nanorod-based photoelectrodes with uSNC coating before (c) and after (d) *J*-t measurement for 10 h (inset: the corresponding electron diffraction pattern of as-prepared photoelectrode).

Ater long-term test, the microstructure of TiO_2_ nanorods fractured with messy HRTEM diffraction spots in the corresponding electron diffraction pattern, indicating that the crystal structure of TiO_2_ photoelectrode was destroyed after measurement. In contrast, the morphology and crystal structure of the uSNC photoelectrode remained unchanged after prolonged operation.


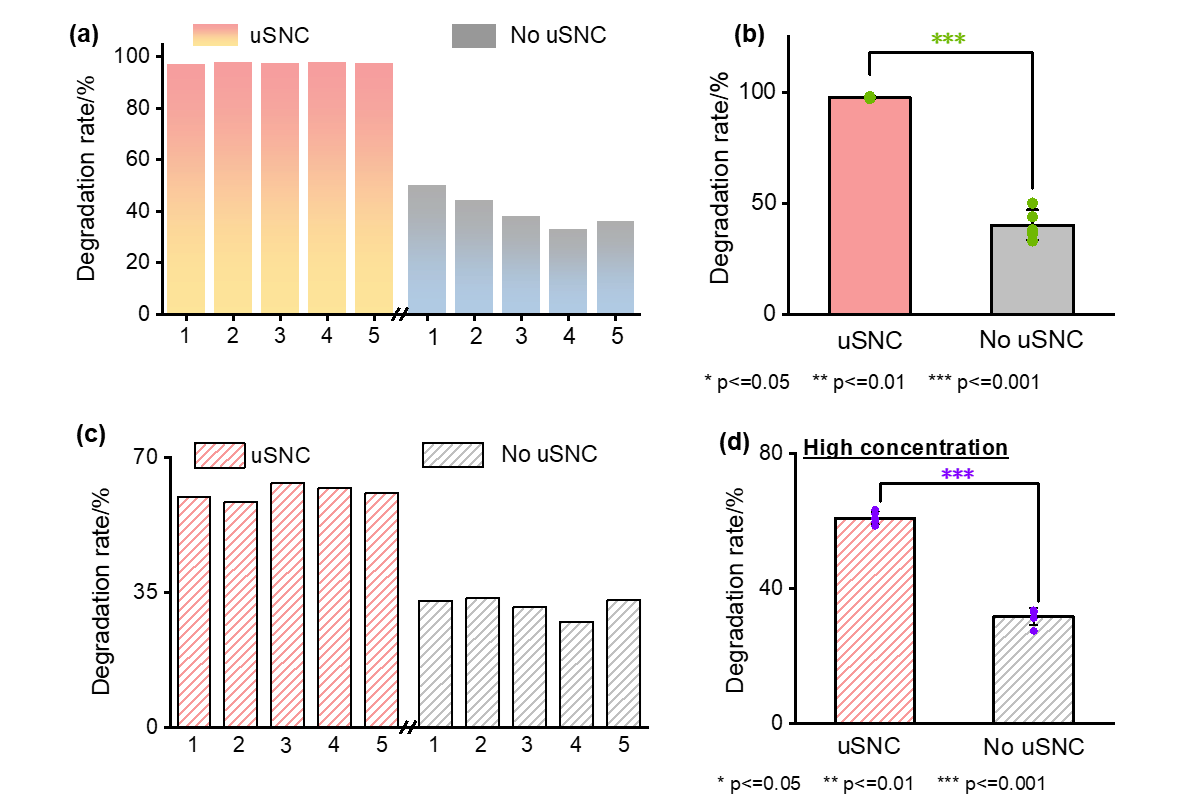


**Figure S15.** (a) Under the UV irradiation, the reproducibility of the prepared photoelectrode with five electrodes for 2 mg L^-1^ MB photoelectrocatalytic degradation after 120 min. (b) Statistics of the MB degradation rate, the error bars represent the standard deviations of five samples. (c) Reproducibility of the prepared photoelectrode with five electrodes for 20 mg L^-1^ MB photoelectrocatalytic degradation after 300 min. (d) Statistics of the MB degradation rate, the error bars represent the standard deviations of five samples. Statistical significance was determined using the two-tailed unpaired Student’s t-test with statistical significance set to: p < 0.05 and results indicated as *p < 0.05, **p < 0.01, ***p < 0.001 and no significance (n.s., p > 0.05).

_
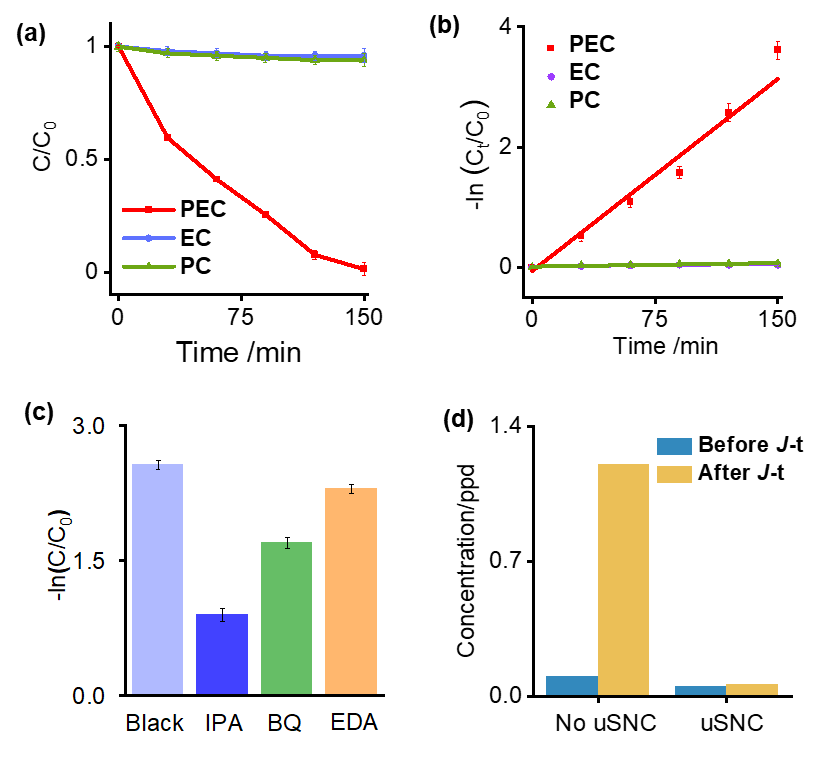
_

**Figure S16.** (a) The degradation rate using uSNC photoelectrode under different processes (PEC, PC, and EC), the error bars represent the standard deviations of triplicate measurements. (b) The pseudo-first-order kinetic curve of uSNC photoelectrode under different processes (PEC, PC, and EC), the error bars represent the standard deviations of triplicate measurements. (c) The quenching experiment used IPA for •OH, BQ for•O_2_^-^ and EDA for h^+^, the error bars represent the standard deviations of three samples. (d) ICP analysis of Na_2_SO_4_ solution for photoelectrodes with and without uSNC coating after degradation MB.

**Figure S16a** showed a comparison between the photocatalysis (PC), electrocatalysis (EC) and PEC processes. The self-degradation of MB was almost negligible under UV–vis light irradiation and the PEC removal efficiency using the TiO_2_ photoelectrode with uSNC (99.7%) was higher than those obtained by PC (30.6%) and EC (10 %), respectively. This observation confirmed that the interfacial electric field produced a large number of photogenerated electrons and holes, which achieved the most effective charge separation efficiency and maximized the use of electrons and holes. The plots of -ln (C_t_/C_0_) versus t of photoelectrode with uSNC were found to be a linear relationship after curve fitting (**Figure S16b**), indicating that the degradation of MB matched pseudo-first-order kinetics. Moreover, **Figure S16c** showed that the •OH and •O_2_^-^ are the major effective active species for MB degradation.

| **Table S1.** Summary of previously reported BiVO_4_, Cu_2_O, MoS_2_, TiO_2_-based photoelectrode for water splitting | | | | | | | | |
| --- | --- | --- | --- | --- | --- | --- | --- | --- |
| Photoelectrocatalysts | Protective method | Thickness of coating | Large apparatus needed | Universality | PEC enhanced value | Stability  duration | *J*/*J*_0_ after the stability duration | Ref. |
| Ov-BiVO_4_@**NiFe-MOFs** | Coating | 15 nm | No | No | 3 | 10 h | 90% | [4] |
| BiVO_4_/FeOOH/NiOOH **in tuning electrolyte** | Tuning electrolyte | — | No | No | 1 | 500 h | 91% | [5] |
| **Cu_3_(BTC)_2_**/Cu_2_O | Coating | 400 nm | No | No | 1 | 400 s | — | [6] |
| TiO_2_/**AZO**/ MoS_2_/Cu_2_O | Coating | 50-100 nm | No | No | — | 5 h | 33% | [7] |
| GaInP_2_-TiO_2_-**MoS_2_/MoO_3_** | Coating | 30 nm | No | No | 1.1 | 20 h | 50% | [8] |
| TiO_2_/PbS-CdS QD/**ZnS** | Coating | — | No | No | 2.4 | 15 h | 60% | [9] |
| TiO_2_/**FeMnP** | Coating | — | Yes | No | 2 | 1.5 h | 80% | [10] |
| n-Si/**TiO_2_/NiOx/pGr** | Coating | — | Yes | No | 1.1 | 12 h | 50% | [11] |
| **PAAM**/Pt/TiO_2_/Sb_2_Se_3_ | Coating | 400 μm | No | **Yes** | 1.2 | 100 h | 70% | [12] |
| **uSNC-TiO_2_** | **Coating** | **5-10 nm** | **No** | **Yes** | **2** | **120 h** | **91%** | **This work** |

**Reference**

[1] Li, Z.; Luo, L.; Li, M.; Chen, W.; Liu, Y.; Yang, J.; Xu, S.-M.; Zhou, H.; Ma, L.; Xu, M.; Kong, X.; Duan, H. *Nat. Commun.* **2021**, 12 (1), 6698.

[2] H. Liu, R. Xie, Y. Luo, Z. Cui, Q. Yu, Z. Gao, Z. Zhang, F. Yang, X. Kang, S. Ge, S. Li, X. Gao, G. Chai, L. Liu, B. Liu, *Nat. Commun.* **2022**, 13, 6382.

[3] X. Wang, W. Wang, J. Liu, J. Qi, Y. He, Y. Wang, W. Hu, Y. Cheng, K. Chen, Y. Hu, A. Mei, H. Han, *Adv. Funct. Mater.* **2022**, 32, 2203872.

[4] Pan, J.; Wang, B.; Wang, J.; Ding, H.; Zhou, W.; Liu, X.; Zhang, J.; Shen, S.; Guo, J.; Chen, L.; Au, C.; Jiang, L.; Yin, S. *Angew. Chem. Int. Ed.* **2021**, 60 (3), 1433–1440.

[5] Lee, D. K.; Choi, K.-S. *Nat. Energy* **2017**, 3 (1), 53–60.

[6] Deng, X.; Li, R.; Wu, S.; Wang, L.; Hu, J.; Ma, J.; Jiang, W.; Zhang, N.; Zheng, X.; Gao, C.; Wang, L.; Zhang, Q.; Zhu, J.; Xiong, Y. *J. Am. Chem. Soc.* **2019**, 141 (27), 10924–10929.

[7] Morales-Guio, C. G.; Tilley, S. D.; Vrubel, H.; Grätzel, M.; Hu, X. *Nat. Commun.* **2014**, 5 (1), 3059.

[8] Gu, J.; Aguiar, J. A.; Ferrere, S.; Steirer, K. X.; Yan, Y.; Xiao, C.; Young, J. L.; Al-Jassim, M.; Neale, N. R.; Turner, J. A. *Nat. Energy* **2017**, 2 (2), 16192.

[9] Kim, T.-Y.; Kim, B. S.; Oh, J. G.; Park, S. C.; Jang, J.; Hamann, T. W.; Kang, Y. S.; Bang, J. H.; Giménez, S.; Kang, Y. S. *ACS Appl. Mater. Interfaces* **2021**, 13 (5), 6208–6218.

[10] Schipper, D. E.; Zhao, Z.; Leitner, A. P.; Xie, L.; Qin, F.; Alam, M. K.; Chen, S.; Wang, D.; Ren, Z.; Wang, Z.; Bao, J.; Whitmire, K. H. *ACS Nano* **2017**, 11 (4), 4051–4059.

[11] Xie, G.; Liu, X.; Guo, B.; Tan, T.; Gong, J. R. *Adv. Mater.* **2024**, 36 (22), 2211008.

[12] Tan, J.; Kang, B.; Kim, K.; Kang, D.; Lee, H.; Ma, S.; Jang, G.; Lee, H.; Moon, J. *Nat. Energy* **2022**, 7 (6), 537–547.
